# Supplementary material for: Prevalence of Chromosomally Located blaCTX-M-55 in Salmonella Typhimurium ST34 Isolates Recovered from a Tertiary Hospital in Guangzhou, China
Source: Microbiol Spectr. 2022 May 26;10(3):e02771-21. doi: 10.1128/spectrum.02771-21 (PMC9241639; doi:10.1128/spectrum.02771-21)
Supplement: SUPPLEMENTAL FILE 2 — Supplemental material. Download spectrum.02771-21-s002.pdf, PDF file, 0.2 MB [file spectrum.02771-21-s002.pdf]

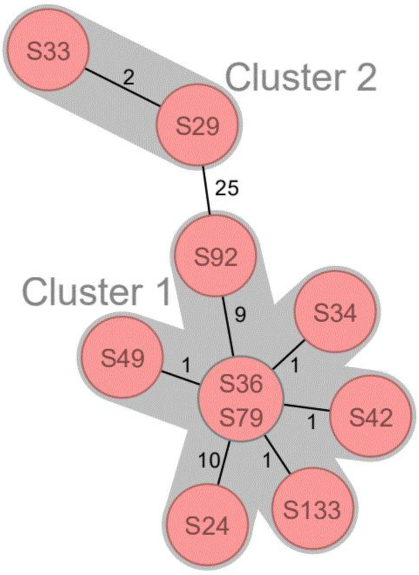

Supplementary Fig. 1 Minimum spanning tree based on cgMLST allele profile of *bla*$_{CTX-M-55}$-positive *S.* Typhimurium ST34 isolates

Each *circle* represents a *bla*$_{CTX-M-55}$-positive *S.* Typhimurium ST34 isolate, whereas the number on each *circle* connecting *line* is the allele difference among the two isolates. Closely related genotypes (the differences of <=10 alleles) are *shaded*, that is, they are divided into a cluster and continuously numbered (1~2).

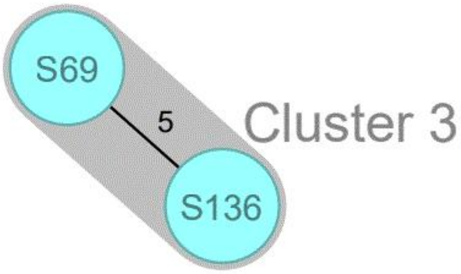

Supplementary Fig. 2 Minimum spanning tree based on cgMLST allele profile of *bla*$_{CTX-M-55}$-positive *S.* Muenster ST321 isolates

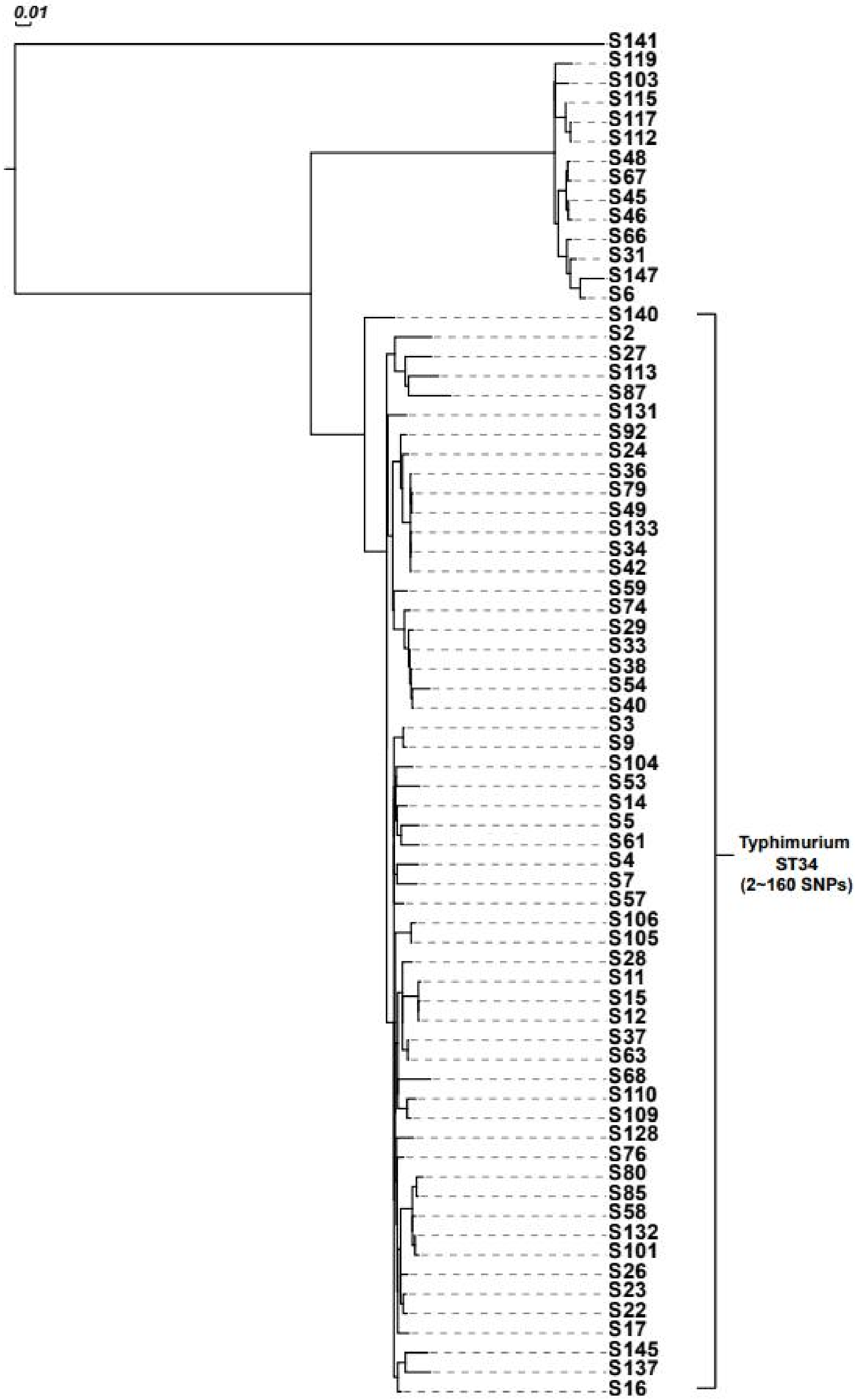

Supplementary Fig. 3 Systematic analysis of *bla*$_{CTX-M-55}$-positive *S.* Typhimurium isolates in this study
